# 3D-Printed PEEK Extravascular Stent in the Treatment of Nutcracker Syndrome: Imaging Evaluation and Short-Term Clinical Outcome

**DOI:** 10.3389/fbioe.2020.00732

**Published:** 2020-07-03

**Authors:** Dali He, Jiahe Liang, Hengen Wang, Yong Jiao, Bin Wu, Dong Cui, Tiesheng Cao, Yanyan Li, Jing Wang, Bo Zhang

**Affiliations:** ^1^Department of Urology, Tangdu Hospital, The Fourth Military Medical University, Xi’an, China; ^2^Department of Ultrasound Diagnostic, Tangdu Hospital, The Fourth Military Medical University, Xi’an, China; ^3^Department of Radiology, Tangdu Hospital, The Fourth Military Medical University, Xi’an, China; ^4^School of Chemical Engineering and Technology, Xi’an Jiaotong University, Xi’an, China

**Keywords:** nutcracker syndrome, 3D printing, polyetheretherketone, extravascular stent, laparoscopy

## Abstract

Minimally invasive options are safe and reliable alternatives for the treatment of nutcracker syndrome (NCS). After continued efforts, our team successfully devised a new and effective therapeutic method: 3D-printed extravascular stenting of the left renal vein. From December 2017 to May 2019, 28 patients (25 men and 3 women) from different parts of China between 18 and 37 years old (mean, 23.6 years) diagnosed with NCS were admitted for laparoscopic 3D-printed extravascular stenting treatment. The post-operative follow-up duration was 6–24 months (median, 16.3 months). Technical success of the operation was achieved in all patients. After treatment, the NCS symptoms all patients resolved or improved during the follow-up period, without relapse. Most symptoms, including macro-/microhematuria, proteinuria, and flank/abdominal pain, tended to resolve within 3–6 months after the surgery; other symptoms, such as left-sided varicocele, also showed varying degrees of improvement at different times post-operatively. Perioperative complications were noted in two patients, including transient and mild lymphatic leakage, without any adverse effects. All extravascular stents were visualized on computed tomography and Doppler ultrasound scans, and no migration or any side effects occurred during the entire follow-up period. Compared to endovascular stenting or polytetrafluoroethylene artificial vessel procedures, 3D-printed polyetheretherketone extravascular stenting has more advantages in terms of stent design and rigidity and approach rationality while successfully preventing stent migration and thrombosis. Therefore, this method may serve as an accurate and effective treatment for NCS patients.

## Introduction

Nutcracker syndrome (NCS), also known as left renal vein (LRV) entrapment syndrome, is characterized by impeded outflow from the LRV into the inferior vena cava due to an abnormally narrow angle between the abdominal aorta (AA) and the superior mesenteric artery (SMA) or between the AA and the spine (Gulleroglu et al., 2014; de Macedo et al., 2018; Park et al., 2018), resembling a nut between the jaws of a nutcracker. This phenomenon results in the formation of venous reflux, renal hilar varices, and an increase in LRV pressure, which mainly cause hematuria, proteinuria, and left flank/abdominal pain, as well as pelvic congestion in females and left-sided varicocele in males. Although uncommon, this diagnosis is important due to the risk of associated morbidities, including chronic kidney disease due to long-term LRV hypertension and LRV thrombosis (Mallat et al., 2013).

At present, the treatment guidelines for NCS are unclear, and different therapeutic principles need to be applied in specific individuals depending on the severity of symptoms. Regardless of the use of a traditional open or minimally invasive approach, the relevant surgical procedures are all designed to relieve LRV hypertension (Dzsinich et al., 2015; Ananthan et al., 2017; Velasquez et al., 2018). Surgical treatment includes laparoscopic surgery, interventional therapy and open surgery. Open surgery options include LRV transposition, gonadocaval venous bypass, renal autotransplantation, and nephropexy (Syed et al., 2015), which have been less reported in recent years due to severe trauma and higher risk. Minimally invasive treatment options include endovascular stenting (EVS), laparoscopic extravascular stenting, laparoscopic splenorenal venous bypass, and laparoscopic LRV transposition for NCS (Chen et al., 2019). EVS and external stenting with expanded polytetrafluoroethylene (ePTFE) artificial vessel interposition around the LRV have been applied relatively recently (Ananthan et al., 2017). Although EVS is a simple and attractive choice, complications including stent migration and thrombosis, though rarely, have been reported (Wei et al., 2003; Chen et al., 2011, 2012). Considering that NCS is mostly due to extrinsic compression of the SMA and preaortic fibrous tissue resulting in LRV entrapment, an appropriate extravascular stent may be effective in relieving LRV stenosis and allowing the recovery of hemodynamics from the outside by supporting the angle between the AA and SMA. Although procedures with ePTFE artificial vessels avoid the potential risks of EVS, the support strength and stability of the stent may still be uncertain due to the simple stent structure. By comparing the advantages and disadvantages of each invasive treatment option, our team successfully devised a new and effective solution: laparoscopic 3D-printed extravascular stenting (3DP-EXVS) of the LRV, a minimally invasive and individualized alternative for treating NCS or posterior NCS (Guo et al., 2018; Wang et al., 2019). Personalized customization of the external stent focuses on fitting to the anatomical characteristics of different individuals to relieve LRV compression while avoiding affecting other important blood vessels and organs.

In recent years, it has become apparent that titanium alloy external stents might not be the optimal option, as they would not only interfere with post-operative imaging results but also increase the risk of magnetic resonance examination. Indeed, a more suitable device should be designed and prepared according to clinical requirements for medical implanting material and due to flaws existing in present titanium alloy external stents. Polyetheretherketone (PEEK) material and improved technologies were used for the development of the new type of external stent, such that the design, weight, mechanical properties and biocompatibility would satisfy the requirements of clinical application. The purpose of this study was to report our experience and to assess the therapeutic value of 3D-printed PEEK extravascular stents in the treatment of NCS over a relatively short follow-up period.

## Materials and Methods

### Eligibility

The diagnosis of NCS relies on clinical manifestations, Doppler ultrasonography (DUS) and computed tomography (CT) results. DUS and CT angiography were performed for all patients for comprehensive assessments of the angle between the AA and SMA, the LRV diameter ratio (hilar to aortomesenteric), the peak velocity (PV) ratio (aortomesenteric to hilar), and hemodynamic characteristics. Sonographic examinations were performed with minimal probe pressure to provide adequate visualization of the LRV, SMA, and AA without causing substantial compression of these vessels. The key criteria for the confirmation of NCS were “the beak sign” (severe form of narrowing of the LRV), the jetting phenomenon and high acceleration of blood flow at the aortomesenteric portion with proximal distention on diagnostic imaging (Orczyk et al., 2017; Kim, 2019). However, there are no standard values for the diagnosis of NCS. The diameter and peak velocity (PV) ratio of the LRV at its narrowed vs. its dilated portion are often used as a diagnostic criterion. An LRV diameter and PV ratio >5 is likely an indicator of NCS, with a sensitivity of 78% and specificity of 100%(Orczyk et al., 2017). In addition, cystoscopy showed blood oozing from the left ureteric orifice in patients with hematuria, urinary cytology was negative for malignancy, and excretory urography revealed no reason for hematuria. Surgical treatment should be considered when conservative therapy has been ineffective for more than 6 months and in patients who exhibit any of the following symptoms: gross hematuria (especially if recurrent), unbearable flank/abdominal pain, severe left-sided varicocele, and persistent moderate or severe proteinuria (Avgerinos et al., 2013). Finally, intraoperative left renal biopsy was routinely performed to determine the degree of renal injury and whether the condition was complicated by other primary glomerulopathies.

From December 2017 to May 2019, 28 patients between 18 and 37 years old diagnosed with NCS were admitted for surgical treatment. The data for the patients undergoing 3DP-EXVS are summarized in Table 1. The measured aortomesenteric angle, LRV diameter ratio and PV ratio were 21.2 ± 4.5 degrees, 6.5 ± 1.1, and 9.6 ± 2.9, respectively, which met the diagnostic criteria.

**TABLE 1:** Demographic and clinical characteristics.

**Variable**	**General information**
**Patient variables**	
No. of patients:	28
N (%):	
Sex	Male, 25 (89.3%)
	Female, 3 (10.7%)
Mean ± SD:	
Age, years	23.6 ± 5.1
Body mass index, kg/m^2^	Male, 19.2 ± 1.4
	Female, 19.0 ± 2.1
**Symptoms**	
N (%):	
Hematuria	Microhematuria, 6 (21.4%)
	Gross hematuria, 11 (39.3%)
Proteinuria*	0.15–1 g/24 h, 8 (28.6%)
	>1 g/24 h, 17 (60.7%)
Flank/abdominal pain^†^	4–6 points, 16 (57.1%)
	7–10 points, 3 (10.7%)
Left-sided varicocele	Grade 2, 1 (3.6%)
	Grade 3, 10 (35.7%)
Chronic fatigue	2 (7.1%)

*24 h urinary protein quantification. ^†^Visual analog scale pain scale.

### Institutional Review

All protocols and informed consent for this treatment program were in compliance with the principles outlined in the Declaration of Helsinki and approved by the Medical Ethics Committee of Tangdu Hospital, The Fourth Military Medical University (no. 2015009, November 2015).

### Preparation of the 3D-Printed Extravascular Stent

All patients underwent 64-row CTA (GE Light Speed VCT) with a slice thickness of 1.0 mm. The anatomical structure was modeled using 3D-DOCTOR software (version 4.0, Able Software Corp., United States). The stent shape and dimensions were designed according to individual imaging data of the target structure using NX software (version 10.0, Siemens, Germany). The extravascular stent consisted of two asymmetrical halves with a hinge in between, forming a closed oval-shaped loop to surround the target blood vessel. The stent has a dumbbell shape that matches the vascular walls of the AA and SMA. Note that the length, curvature and lateral edges of the stent on the AA side should be carefully designed to avoid compression of the renal arteries. We usually measure the maximum cross-sectional area of the distally dilated LRV to estimate the space to be reserved inside the stent. Additionally, in children, growth/enlargement of the blood vessel should be taken into account in the design of the stent, as it is necessary to leave proper space for blood vessel growth. The wall thickness of the stent is approximately 1.5 mm. The inner diameter (including the major axis and minor axis) of the stent is approximately 1.0–1.5 cm, fully permitting normal passage of the LRV. Moreover, the stent contains multiple holes in the sides to reduce its weight and provide space for adhesion to the surrounding tissue to effectively prevent migration and, more importantly, allow hemodynamic changes in the vessel within the stent to be accurately measured by DUS. During the process, mechanical design aspects were considered fully. By means of biomechanical finite element analysis, the maximum deformation, maximum stress, and fatigue period of the stent can be obtained by setting the limit loading conditions based on the surrounding environment to ensure that the mechanical properties meet the clinical requirements.

**FIGURE 1:**
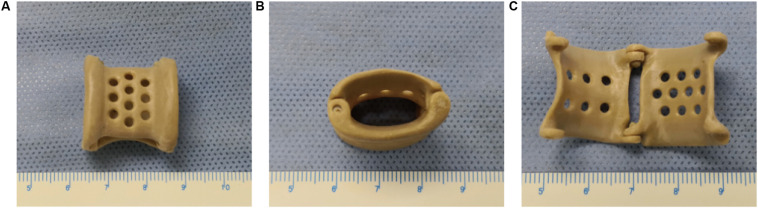
The overall appearance of the 3D-printed polyetheretherketone extravascular stent (weight, approximately 1.8 g). **(A)** Anterior view. **(B)** Lateral view. **(C)** Internal view.

After the stent design was completed, the modeling data were input into a Fused Deposition Modeling 3D printer (FUNMAT PRO 410, INTAMSYS) that used PEEK material (770G; Food and Drug Administration registration number: 19532334806) to generate the product (Figure 1). Ultrasonic cleaning, ethylene oxide sterilization, and disinfection monitoring were performed before the stent was implanted.

### Surgical Procedures

**FIGURE 2:**
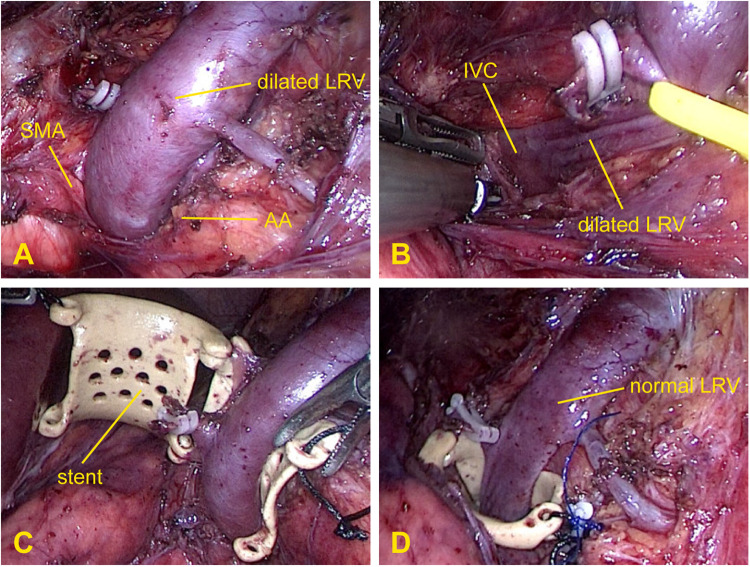
Intraoperative photographs demonstrating the surgical procedure for extravascular stenting. **(A)** Exposure of the dilated left renal vein and gonadal vein and resection of the adrenal vein. **(B)** Release of the preaortic fibrous ring between the aorta and superior mesenteric artery. **(C)** Placement of the stent around the left renal vein. **(D)** Fixation of the stent to the surrounding fibrous tissue where the left renal vein was compressed and immediate relief of the venous engorgement.

All operations were successfully performed in the operating room. After general anesthesia was administered, the patient was positioned in a modified flank position, with the left side facing upward. The operation was performed by a three-port laparoscopic technique through a transabdominal approach. The camera port was located at the left border of the rectus muscles approximately 5 cm from the umbilicus; the first operating port was placed in the upper-left quadrant, and the second was placed at the left midclavicular line below the costal margin. During the operation, the pneumoperitoneum pressure was maintained at 12–13 mmHg. Under direct vision, the retroperitoneum was cut open along the left paracolic sulcus; the LRV, which was markedly dilated, as well as the left gonadal vein and adrenal vein draining into the LRV, were identified (Figure 2A). The fibrous ring between the AA and SMA was then divided, and the LRV was dissected medially from the renal hilum to the inferior vena cava until it was completely mobilized (Figure 2B). The PEEK extravascular stent was inserted into the body through the camera port and then placed around the compressed portion of the LRV (Figure 2C). When correct positioning of the stent was verified, the stent was fixed firmly to the surrounding fibrous tissue at the distal edge of the stent with a non-absorbable 4.0 Prolene suture to prevent slippage (Figure 2D). The left gonadal vein and adrenal vein were usually left in place; however, in some cases, they were resected because of difficulties with stent placement or severe varicocele. After the operation, no special post-operative treatment was required except for standard anti-inflammatory therapy and fluid supplementation.

### Follow-Up

The post-operative follow-up duration was 6–24 months (median, 16.3 months). Routine clinical and imaging examinations were scheduled at 1 week, 3 months, 6 months, and 12 months and then annually thereafter unless symptoms recurred. Because DUS results are somewhat subjective, to minimize errors, all patients were examined by the same experienced ultrasound physician. Relapse was defined as the recurrence of at least one symptom associated with NCS.

### Statistical Analysis

All statistical calculations were performed with SPSS software, version 16.0 for PC (SPSS, Inc., United States). Paired-samples *t*-tests were performed to compare patient data at different times before and after surgery. A *P*-value < 0.05 was considered significant.

## Results

**FIGURE 3:**
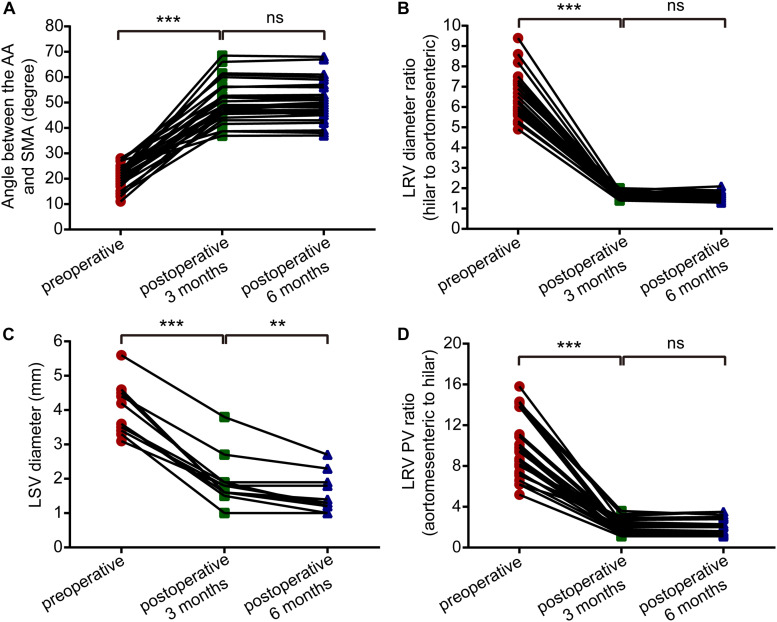
Paired-samples *t*-tests showed differences between pre-operative and post-operative imaging parameters (pre-operative vs. post-operative 3 months; and post-operative 3 months vs. post-operative 6 months) in patients with nutcracker syndrome. **(A)** Angle between the abdominal aorta and superior mesenteric artery. **(B)** The left renal vein diameter ratio (hilar to aortomesenteric). **(C)** The left spermatic vein diameter. **(D)** The left renal vein peak velocity ratio (hilar to aortomesenteric). ***P < 0.001, **P < 0.01, ns = not significant.

CT and DUS examinations are very important for patients with NCS, and we often use imaging data combined with clinical evaluations to assess a patient’s condition. For this study, we compared the imaging results from the 3-month follow-up with pre-operative indexes (Table 2A). CTA showed that the angle between the AA and SMA increased from 21.2 ± 4.5 degrees to 50.3 ± 8.0 degrees (difference: 29.1 ± 8.2 degrees, *P* < 0.001) and that the anteroposterior diameter ratio of the LRV (hilar to aortomesenteric) decreased from 6.5 ± 1.1 to 1.7 ± 0.2 (difference: 4.9 ± 1.0, *P* < 0.001). In patients with varicocele, DUS indicated that the left spermatic vein (LSV) diameter decreased from 4.0 ± 0.7 mm to 1.9 ± 0.7 mm on natural breathing (difference: 2.1 ± 0.5 mm, *P* < 0.001). According to hemodynamic results, the blood flow within the stent was unobstructed, and the PV ratio (aortomesenteric to hilar) decreased from 9.6 ± 2.9 to 1.9 ± 0.7 (difference: 7.7 ± 2.8, *P* < 0.001). By comparing the imaging data at 3 and 6 months post-operatively (Table 2B), we observed that patients with left varicocele still exhibited improvement in the LSV diameter, which decreased from 1.9 ± 0.7 mm to 1.6 ± 0.6 mm (difference: 0.4 ± 0.3 mm, *P* < 0.05), though differences in the other three variables (aortomesenteric angle, LRV diameter ratio and PV ratio) were not significant (*P* > 0.05). All differences between pre-operative and post-operative imaging parameters in patients with NCS are provided in Figure 3. Although the follow-up period was different for each patient, the current observations indicate that the imaging parameters of most patients stabilized within 3 months after the surgery, with the possibility of further improvement in LSV diameter in those with left-sided varicocele.

**TABLE 2A:** Comparison of the pre-operative and 3-month post-operative imaging parameters.

**Variables**	**N**	**Pre-operative**	**Post-operative 3 months**	**Difference**	***t***	**Sig. (2-tailed)**
Angle between the AA and SMA (degree)	28	21.2 ± 4.5	50.3 ± 8.0	−29.1 ± 8.2	−18.681	0.000
LRV diameter ratio (hilar to aortomesenteric)	28	6.5 ± 1.1	1.7 ± 0.2	4.9 ± 1.0	25.109	0.000
LRV PV ratio (aortomesenteric to hilar)	28	9.6 ± 2.9	1.9 ± 0.7	7.7 ± 2.8	14.810	0.000
LSV diameter (mm)	11*	4.1 ± 1.2	2.2 ± 1.0	1.9 ± 0.8	9.129	0.000

Paired-samples t-test (α = 0.05). *Left-sided varicocele was found in 11 of the 28 patients.

**TABLE 2B:** Comparison of the 3- and 6-month post-operative imaging parameters.

**Variables**	**N**	**Post-operative 3 months**	**Post-operative 6 months**	**Difference**	***t***	**Sig. (2-tailed)**
Angle between the AA and SMA (degree)	28	50.3 ± 8.0	50.4 ± 8.0	−0.1 ± 0.9	−0.642	0.526
LRV diameter ratio (hilar to aortomesenteric)	28	1.7 ± 0.2	1.7 ± 0.2	0.0 ± 0.1	0.214	0.832
LRV PV ratio (aortomesenteric to hilar)	28	1.9 ± 0.7	1.9 ± 0.7	−0.0 ± 0.1	−0.130	0.897
LSV diameter (mm)	11*	2.2 ± 1.0	1.6 ± 0.6	0.4 ± 0.3	3.691	0.004

Paired-samples t-test (α = 0.05). *Left-sided varicocele was found in 11 of the 28 patients.

**FIGURE 4:**
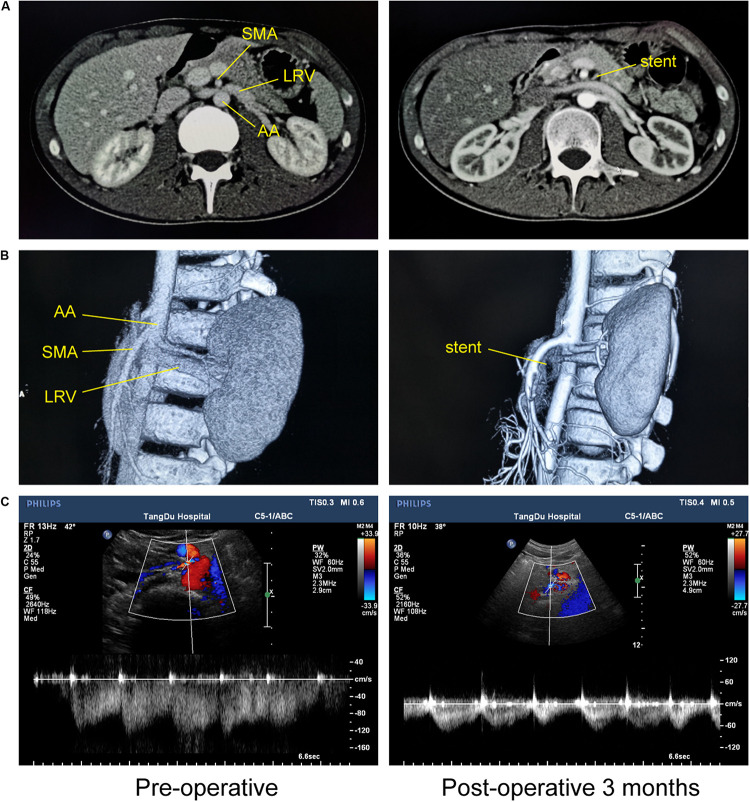
Imaging comparisons before and at 3 months after 3D-printed extravascular stenting. **(A)** Computed tomography. **(B)** 3D computed tomography reconstruction. **(C)** Doppler ultrasound.

Overall, the pre-operative and post-operative imaging results were significantly different, and we even found that venous engorgement immediately subsided after extravascular stenting during the operation. Although post-operative imaging data remained constant, we also found that LRV compression was eliminated due to the presence of the extravascular stent and that the blood flow within the stent was unimpeded (Figure 4). All these dramatic changes revealed that LRV entrapment was effectively relieved. Moreover, we compared the major vessels adjacent to the stent before and after surgery, including the SMA, AA, and the left and right renal arteries. No obvious deformation was observed, demonstrating that the extravascular stent did not compress the surrounding vessels, fully embodying the advantages of individualized customization.

Technical success of the operation was achieved in all patients. The mean operation time, bleeding volume, and post-operative length of stay were 70.8 min (range, 50–90 min), 30.2 ml (range, 20–40 ml), and 7.3 days (range, 6–8 days), respectively. Outcomes were classified as excellent (complete symptom resolution), good (significant symptom improvement), and poor (no symptom improvement or worsening), and the results are given in Table 3. All patients showed a significant improvement during follow-up, and most NCS symptoms tended to subside within 3–6 months after the surgery, especially abdominal pain and fatigue, which were relieved in a short time. Among the 11 patients with varicocele, 7 had LSV diameters that returned to nearly normal with natural breathing within 12 months post-operatively; the other four consistently showed signs during the entire follow-up period but had much better diameters than before the operation. Of the 17 hematuria patients, macro-/microhematuria was completely resolved within 1 week, 3 weeks and 12 months post-operatively in 4 patients, 10 patients, and 1 patient, respectively. Two patients showed occult blood in the urine and abnormal urinary protein at each review, and the renal biopsy results of both demonstrated definite IgA nephropathy. Nonetheless, we observed continued improvement in these two patients, whereby the gross hematuria with blood clots gradually progressed to microscopic hematuria, with a marked reduction in red blood cells in the urine after stenting, combined with the disappearance of abdominal pain. In addition, for most of the 25 cases of proteinuria, improvement or recovery occurred in the early post-operative period, and complete remission of the symptoms was observed in 23 cases. Perioperative complications were noted in two patients, including transient and mild lymphatic leakage, though without any adverse effects. All extravascular stents in the patients were visualized on CT and DUS scans, and no migration or other complications occurred during the follow-up period.

**TABLE 3:** Clinical symptoms in patients with NCS after 3DP-EXVS.

**Symptoms**	**Pre-operative**	**Post-operative**		
		**1 week**	**3–12 months**	**12–24 months**^†^
N:				
Macro/microhematuria	17	13	3	2*
Proteinuria	25	11	4	2*
Flank/abdominal pain	19	19	2	0
Left-sided varicocele	11	11	7	4*
Chronic fatigue	2	2	0	0

*Symptom persisted during the follow-up period. ^†^Outcome: excellent in 20 patients; good in 8 patients; poor in 0 patients.

## Discussion

NCS is relatively rare, but an increasing number of explorations have been conducted on this condition. Although the guidelines for treating NCS continue to be debated, the consensus is that invasive treatment should be considered for those in whom conservative therapy fails and in those exhibiting persistent severe symptoms or worsening laboratory/imaging results (Xu et al., 2009; Daily et al., 2012; Avgerinos et al., 2013; Berthelot et al., 2017). Although renal impairment may not be significant in the early stages of NCS, prompt and effective treatment may prevent further deterioration caused by the disease (Denchev et al., 2018). Traditional renovascular surgery is associated with a high surgical morbidity rate and several complications, such as venous thrombosis, cardiovascular accidents, anastomotic bleeding and restenosis (Hohenfellner et al., 2002; Reed et al., 2009; Avgerinos et al., 2013); thus, it is almost never performed in the modern era. EVS is an attractive and simple minimally invasive option that has a positive therapeutic effect in NCS. However, the risk of complications should not be overlooked, especially of stent migration, which is noted in 7.3% of all cases and is likely to cause thrombosis, vessel trauma and embolization (Wei et al., 2003; Chen et al., 2011, 2012). A larger study of EVS in 75 patients reported a stent migration rate of 6.7%, with ultimate migration from the LRV into the inferior vena cava or the heart (Wu et al., 2016). Furthermore, long-term antiplatelet therapy is recommended after endovascular stenting while the stent becomes endothelialized (He et al., 2014). Although the outcomes of EVS treatment are encouraging, it is not a permanent solution for NCS, and for this reason, safer and more logical and effective surgical procedures may provide better outcomes.

In recent decades, ePTFE artificial vessels have been used as extravascular stents for NCS because they involve minimal trauma, few complications, and a simple operation (Zhang et al., 2010; Li et al., 2014; Wang et al., 2015; Sorokin et al., 2018; Chen et al., 2019). Barnes et al. (1988) performed the first ePTFE graft placement via an open approach in 1988. The first reported laparoscopic placement was performed in the United States in 2001, and in light of the potential risks of EVS, this strategy has been recommended as a better option for NCS (Scultetus et al., 2001). In 2015, Wang et al. (2015) reported their experience with the use of ePTFE artificial vessels. In certain cases, they chose externally reinforced ePTFE grafts that matched or were longer than the length of the LRV from the gonadal vein to the inferior vena cava. They reported that symptoms resolved in 10 patients and improved in 2, though one patient developed recurrent gross hematuria because of stent migration. Tian et al. (2015) first reported one case of extravascular stent management that acted as a remedial measure for the migration of LRV endovascular stents in NCS. Importantly, however, these artificial blood vessels were not designed to serve as external vascular stents, and the single design hardly matches the diversity of anatomical features of the compressed portion of the LRV among individuals. In addition, the stents were split lengthwise before being wrapped around the vessel, which might affect both the strength and stability of the stent, and there are still no detailed descriptions of the fixation method for this technique in the literature. Furthermore, the possibility of graft deformation and migration and restenosis of the LRV needs to be investigated. To solve these problems, our team proposed 3DP-EXVS for the treatment of NCS; this extravascular stent exhibits good biocompatibility and static mechanical properties; moreover, personalized customization and stable fixation can be achieved, which together result in permanent decompression of the LRV and relief of the corresponding symptoms. Using this technique, we achieved significant success in all patients.

Currently, successful applications of 3D printing have been reported in the field of surgery, and the greatest advantage of 3D printing is that artificial implants can be customized accurately and effectively (Pugliese et al., 2018). Our study attempted to address the problem of LRV stenosis using 3D-printed extravascular stents because custom-made implants better match target structures in the body. To prevent compression of the surrounding major vessels, the stent shape should not only be matched to the vascular walls of the AA and SMA but should also be trimmed to leave space for the left and right renal arteries, especially at the two ends. The choice of material is also equally important. The safety of biomedical materials is mainly reflected by the interaction between the tissues and materials, and to meet the standards of implantable devices, biomedical materials must have an acceptable response after implantation in the human body, without qualitative changes in structure or performance. At present, PEEK surgical implants are widely used in clinical practice, and research on biocompatibility continues to advance our knowledge (Panayotov et al., 2016). Our team selected PEEK because it not only has the same mechanical properties as titanium alloy but also has a density similar to that of the human body, resulting in good cell viability and proliferation *in vivo* (da Cruz et al., 2019), with fewer adverse effects due to external acceleration and centrifugal forces. In fact, one of the 28 patients, a young pilot who received treatment with robot-assisted 3DP-EXVS, has already resumed his duties as a pilot, without symptoms or discomfort under the high acceleration and centrifugal forces experienced in an airplane (Wu et al., 2019).

As a particular type of minimally invasive vascular surgery, 3DP-EXVS has shown satisfactory reliability and efficacy. Certain surgical risks can be greatly reduced by careful dissection and rich surgical experience. Lymphatic leakage was observed in a small number of patients, with an incidence of 7.1% (2/28), and it was associated with intraoperative damage to lymphatic vessels during vascular dissociation. In general, this complication is relatively mild and self-limited, and accurate intraoperative bipolar hemostasis and appropriate extension of the duration of indwelling catheterization can effectively resolve the issue. Compared to other surgical options, 3DP-EXVS shows advantages in terms of fewer side effects and complications. Traditional open surgery causes great surgical trauma and carries high risks, EVS may result in venous thrombosis, in-stent restenosis and stent migration, and the single design of ePTFE grafts is hardly able to match the anatomical variations of the LRV among individuals. In contrast, 3D-printed PEEK external stents have more advantages in terms of stent design, weight, rigidity and approach rationality while successfully preventing thrombosis, stent deformation and migration, without changing the anatomical structure or damaging the LRV intima.

In our study, 28 patients diagnosed with NCS were admitted for 3DP-EXVS treatment. The symptoms of all patients resolved or improved during the follow-up period, without relapses, suggesting that renal injury caused by NCS may be a reversible chronic process when pathological results show minor kidney damage. Furthermore, the positive effects of the surgery were demonstrated even when combined with IgA nephropathy in some cases.

According to the current study, 3DP-EXVS yields good results in the treatment of NCS. With the help of 3D printing technology, this treatment method is more accurate and personalized than others and can be applied to all patients who meet the surgical indications, including both adolescents and adults in whom conservative treatments have failed, or can even serve as a remedy for failed interventional therapy. We aimed to obtain the best therapeutic effect with a minimum level of trauma and complications, and 3DP-EXVS is undoubtedly one of the best options. Nevertheless, additional studies are necessary to conclude that this technique has a satisfactory effect in contemporary practice.

## Data Availability Statement

All datasets generated for this study are included in the article/supplementary material.

## Ethics Statement

All protocols and informed consent for this treatment program were approved by the Medical Ethics Committee of Tangdu Hospital, the Fourth Military Medical University (No. 2015009, November 2015). The patients/participants provided their written informed consent to participate in this study.

## Author Contributions

DH, JL, and BZ contributed to the conception and design of the study, discussion of the results, and drafting of the manuscript. YJ, BW, and BZ were responsible for the diagnosis and treatment of the disease. HW and DC organized the follow-up research. YL and TC performed the imaging examinations and data collection. JW and BZ carried out the 3D modeling, data post-processing, and stent manufacturing. All authors contributed to the article and approved the submitted version.

## Conflict of Interest

The authors declare that the research was conducted in the absence of any commercial or financial relationships that could be construed as a potential conflict of interest.

## Abbreviations

NCS: nutcracker syndrome
LRV: left renal vein
AA: abdominal aorta
SMA: superior mesenteric artery
EVS: endovascular stenting
ePTFE: expanded polytetrafluoroethylene
3DP-EXVS: 3D-printed extravascular stenting
PEEK: polyetheretherketone
DUS: Doppler ultrasound
CT: computed tomography
PV: peak velocity
LSV: left spermatic vein.
